# Minimizing Early-Onset Lymphedema Following Groin Dissection in Metastatic Melanoma

**DOI:** 10.1245/s10434-026-19559-4

**Published:** 2026-04-13

**Authors:** Melody Brown, Teresa S. Lee, Isabel Li, Christopher Allan

**Affiliations:** 1 Mater Hospital, Brisbane, Australia; 2 Royal North Shore Hospital, St Leonards, Australia; 3 University of Sydney, Camperdown, Australia; 4 NHMRC Clinical Trials Centre, Sydney, Australia

## Abstract

**Background:**

Lymphedema is a common and burdensome complication after groin dissection for metastatic melanoma. This study evaluated whether a 6-month program of compression garments combined with simple lymphatic drainage (CG-SLD) reduces the rate of lymphedema presentation compared with standard care (SC).

**Patients and Methods:**

Participants were randomized 1:1 to SC or CG-SLD for 6 months postoperatively. Lymphedema was assessed preoperatively and every 3 months for 24 months using interlimb volume difference and bioimpedance spectroscopy. The primary end point was the incidence of lymphedema at 24 months. Secondary outcomes included time to lymphedema development, lymphedema severity, and quality of life (QoL). The study was powered to detect a reduction in 24 month incidence from 45 to 18% (*α* = 0.05, 80% power), requiring 88 participants.

**Results:**

A total of 38 participants were randomized (SC *n* = 20; CG-SLD *n* = 18), below the planned sample size. At 24 months, lymphedema incidence was numerically higher but nonsignificant for SC compared with CG-SLD; 55% (31.5–76.9) versus 38.9% (17.3–64.3). All new lymphedema events occurred within 12 months. Early lymphedema severity at 3 months favored CG-SLD; however, no persistent between-group differences were observed at later time points. There was no statistical evidence to support a difference in QoL scores at any time point.

**Conclusions:**

CG-SLD did not reduce lymphedema incidence compared with SC at 24 months. Although early reductions in lymphedema incidence and severity were observed for CG-SLD, these were not maintained beyond 6 months when interventions ceased. The study was underpowered, and larger trials are required to determine whether early prophylactic strategies provide durable benefit.

**Supplementary Information:**

The online version contains supplementary material available at 10.1245/s10434-026-19559-4.

Regional lymph node relapse in the groin is the most common site of recurrent disease following excision of a primary melanoma from the lower limb and lower torso. The risk is directly proportional to the thickness of the primary lesion and for intermediate thickness lesions (1–4mm), ranges from 8 to 30%. The morbidity of groin dissection, including inguinal and ilioinguinal lymphadenectomy is significant, and in the short-term can include wound infection, wound breakdown, prolonged lymph drainage and seroma, while lymphedema predominates in the longer term.^1–3^

Lymphedema is a much-feared side-effect of lymphadenectomy resulting in persistent high protein edema in the interstitial tissues of the affected limb. Lymphedema is a chronic, incurable condition, but may be alleviated by appropriate management. If ignored, lymphedema can progress and become difficult to manage.^4^ The risk of lymphedema following groin dissection for melanoma is significant although the reported rates are quite varied. A meta-analysis of 20 studies in 2016 found leg lymphedema developed in approximately 33% (25%–42%) of patients who underwent groin lymphadenectomy for melanoma.^5^ Primarily, this is a function of inconsistent definition and classification of lymphedema, but it may also be affected by variations in surgical technique, radiation, wound complications, and comorbidities such as obesity and heart failure.^6,7^

The morbidity of lymphedema is both physical and psychological. The physical morbidity includes feelings of limb tightness, heaviness and pain, increased risk of cellulitis, and reduced function and mobility.^3^ Psychologically, lymphedema may result in distorted body image, depression, and anxiety.^8^ Additionally, lymphedema can result in significant financial burden on those affected owing to treatment and compression garment expenses, time off work and sometimes, the need to change occupations.^9^

The extent of preventative action against lymphedema development may be a factor that could help to reduce the overall burden. Practice varies in this regard, with the traditional position being to intervene only if lymphedema develops. A more proactive approach is now favored and there is good evidence from breast cancer-related lymphedema research that support early detection and intervention for overall reductions of lymphedema incidence in the long term.^10^ The active lymphedema prevention strategies used in these breast cancer trials were compression garments^11,12^ and manual lymphatic drainage to a lesser extent.^13,14^

There has been a paucity of studies evaluating the role of lymphedema compression garments in patients with melanoma after lymphadenectomy. In 2013, there was a randomized controlled trial in a mixed melanoma/urogenital cancer cohort that found prophylactic compression garments to be ineffective in the prevention of leg lymphedema after inguinal or ilio-inguinal lymphadenectomy in patients up to 12 months after surgery.^15^ The authors reported a 14% relative risk reduction in lymphedema among patients who wore compression stockings during the first 6 months after surgery, although this finding did not reach statistical significance. It is possible that a longer follow-up would increase the strength of this finding. An incidental finding from another melanoma trial with a comparable cohort, reported a 39% reduction in the odds of leg lymphedema among patients who wore compression garments at any time during the first 2 years following groin dissection.^6^ As compression garment use was not a prespecified intervention, it was not possible to determine whether the observed benefit represented prophylaxis or treatment of established lymphedema.

The primary objective of this study was to determine if prophylactic lower limb compression garments and simple lymphatic drainage (CG-SLD) implemented for a period of 6 months postoperatively reduces the rate of lymphedema at 2 years compared with standard care (SC). Secondary objectives of the study were to determine the severity of lymphedema and quality of life (QoL) of both groups.

## Patients and Methods

### Study Design and Participants

Patients undergoing inguinal or ilioinguinal lymphadenectomy for metastatic melanoma were identified and enrolled at the time of surgical booking at Mater Adult and Mater Private Hospitals, Brisbane. Patients were included if they met the following inclusion criteria: aged 18 years and over with confirmed metastatic melanoma to the groin, and were undergoing inguinal or ilioinguinal lymphadenectomy, were able to give informed consent and attend regular follow-up, and had the ability to apply and remove (by self, partner or carer) compression shorts and full-length leg compression garment. Patients were excluded if they had peripheral arterial disease (Ankle Brachial Pressure Index < 0.6), pre-existing lymphedema, bilateral groin dissection, previous contralateral groin dissection, infection, or open wound at any site in contact or adjacent to the compression stocking, acute cellulitis in the affected leg, acute deep venous thrombosis in the affected leg, known allergy to compression stocking material, and/or previous severe skin reactions to compression stockings. Ethnicity of our participants was not collected because it was not a required variable at the time of the study.

The study commenced in 2010, prior to local requirements for prospective registration of noncommercial investigator-initiated surgical trials. The protocol, including predefined primary and secondary outcomes, was approved by the Mater Health Services Human Research Ethics Committee before recruitment commenced.

### Interventions

Participants were randomly allocated to either the SC or CG-SLD groups via the Mater Adult Hospital Research Support Centre randomization program at the time of recruitment. Randomization was 1:1, with equal chance of being in the SC or CG-SLD group of the trial. The SC group were given standard management which consisted of an exercise and mobility program, education on postoperative activity recommendations and lymphedema precautions by an accredited lymphedema-trained occupational therapist or physiotherapist prior to hospital discharge. The CG-SLD group also received the same education and postoperative care as the SC group but were provided with additional instruction on wearing compression garments and performing simple lymphatic drainage. Both groups were assessed and instructed by Australian Health Practitioner Regulation Agency (AHPRA) registered lymphedema therapists to ensure best practice was maintained for the study duration.

Specifically, those in the CG-SLD group were fitted with compression garments after the surgical drains were removed, or at the completion of radiation. Compression shorts (Skins^TM^ 17 mmHg) and a ready-to-wear chap-style or thigh-length compression garment (20–30 mmHg) were prescribed by a lymphedema therapist. Participants were instructed to wear the combination of compression shorts and thigh-high garments daily during the daytime (for at least 12 h of the day). The compression garments were removed for hygiene/showering, wound care, and sleeping each night. Participants in the CG-SLD group were instructed in simple lymphatic drainage that could be self-administered by the participant or their support person once per day. The garment prescription and fitting, as well as instruction and review for simple lymphatic drainage involved four 30-min, 1:1 education sessions with the lymphedema therapist. CG-SLD was continued for 6 months postoperatively. If no lymphedema was detected at 6 months, CG-SLD was gradually weaned over the subsequent month. Any participant deemed to have developed lymphedema during follow-up was recommended to commence lymphedema treatment irrespective of their randomized group, according to standard practice of compression bandaging/garments, manual lymphatic drainage, exercise, and other treatment modalities.^4^

### Outcome Measures

Lymphedema volumes were calculated on the basis of leg circumferences, taken 10 cm apart, as per the Australasian Lymphology Association Guidelines.^16^ These circumferences were then entered into a software package - Limb Volumes Professional (LVP 5.0), for calculation of Inter-limb Volume Difference (ILVD) via a truncated cone formula. Bioimpedance spectroscopy (Impedimed U400®) was also used to account for extracellular fluid changes in the at-risk leg. The resistance and reactance to current flow in the extracellular fluid of the operated leg was compared with the contralateral side via use of surface skin electrodes, producing a Lymphedema Index (L-Dex®). Bioimpedance spectroscopy is unaffected by changes in the participant’s weight owing to a change in fat mass or muscle mass and provides an instant tool for assisting in the clinical assessment of lymphedema change.

Lymphedema was considered present when L-Dex® ≥ 10 compared with baseline and ILVD was > 10% compared with the unaffected limb. These thresholds were based on the International Lymphedema Framework Best Practice Consensus Report^4^ and the few published clinical trials available for lower limb lymphedema at the time of study.^7,8^

EORTC QLQ-30 and LYMQOL (leg) questionnaires were administered to evaluate QoL in our participants. The LYMQOL (leg) comprises of 22 items across four domains: function, appearance, symptoms, and mood, as well as a single overall QoL item.^17^

All measurements were taken by blinded assessors who were unaware of the participant group allocation or previous measurements. Participants in the CG-SLD group were instructed to remove compression garments 1 h prior to arrival for measurement, to minimize the visibility of marks left by the compression garment on their skin.

Measures were performed preoperatively and at follow-up every 3 months for 2 years, starting after removal of drains or at the end of radiation. Follow-up visits coincided with routine surgical outpatient follow-up wherever practicable.

### End Points

The primary end point was the proportion of participants who presented with lymphedema (at least once) in each group at 24 months after surgery. Secondary end points included time to lymphedema development, lymphedema severity, and health-related QoL over 24 months.

### Sample Size and Statistical Analysis

The sample size was calculated to detect a reduction in lymphedema incidence from 45% to 18% at 2 years of follow-up, based on rates reported in previous studies.^7,18^ Assuming a two-sided test with Type 1 error of 0.05 and 80% power, 88 participants (44 per group) were required.

An intention-to-treat approach was applied for all analyses. Descriptive statistics were generated to summarize demographic and clinical characteristics within each treatment group. Associations between treatment groups and lymphedema outcomes were assessed using Fisher’s exact test for categorical variables. Severity of lymphedema was compared between groups at time points using Welch’s two sample *t*-tests. Time-to-lymphedema development was analyzed using Kaplan–Meier estimator, with group differences compared using the log-rank test. Cumulative incidence functions accounting for death as a competing risk were evaluated using Gray’s test. Raw EORTC scores were calculated and linearly transformed to a 0–100 scale in accordance with the EORTC scoring manual.^19^ Minimally Important Difference (MID) of ≥ 10 points between-group^20^ and Thresholds of Clinical Importance (TCI)^21^ were used for interpretation of mean scores. LYMQOL domain scores were calculated as the mean of item responses, with scores ranging from 1 to 4, where lower scores indicate fewer problems. LYMQOL Global QoL score ranged from 0 to 10, with higher scores indicating better QoL.^17^

## Results

**Fig. 1:**
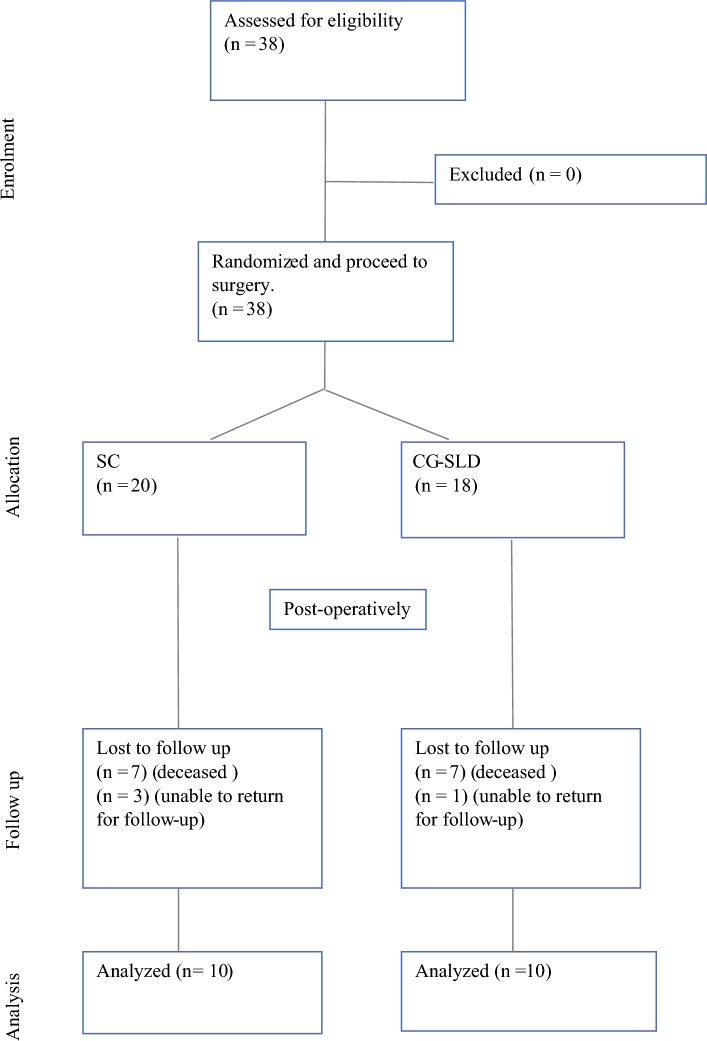
Consort diagram

This study recruited a total of 38 participants (20 in the SC group and 18 in the CG-SLD group) between September 2010 and September 2015. Our sample did not meet our planned target recruitment over this 5 year period. This was owing to the falling rates of lymph node dissection following the widespread adoption of sentinel node biopsy and the advancement of systemic therapies.^2, 22^ Of the 38 patients who had inguinal or ilio-inguinal lymphadenectomy for melanoma, all were eligible to be randomized preoperatively. There was a high mortality rate during follow-up, which resulted in a total of 20 participants available for follow-up at 2 years (Fig. 1).

### Baseline, Clinical, and Post-Operative Characteristics

Baseline characteristics were well-balanced between groups. The mean age was 54.6 years (SD 14.4), and the mean BMI was 29.2 (SD 6.6). Gender distribution was not significantly different (*p* = 0.10). Dominant limb involvement was similar (*p* = 0.7) (Table 1).

**Table 1:** Baseline, clinical and post-operative characteristics

		Overall *N* = 38	SC *N* = 20	CG-SLD *N* = 18	*p*-value
Age	Mean (SD)	54.6 (14.4)	54.7 (24.9)	54.5 (14.3)	> 0.9
Gender	Female	14 (27%)	10 (50%)	4 (22%)	0.10
	Male	24 (63%)	10 (50%)	14 (78%)	
BMI	Mean	29.2 (6.6)	28.9 (7.7)	29.5 (5.3)	0.8
Dominant limb affected	*N* (%)	15 (39%)	7 (35%)	8 (44%)	0.7
Type of surgery	Inguinal	1 (2.6%)	1 (5.0%)	0 (0%)	> 0.9
	Ilio-inguinal	37 (97%)	19 (95%)	18 (100%)	
No. of lymph nodes removed	Mean (SD)	20.3 (7.0)	18.5 (6.6)	22.3(7.0)	0.1
No. Of positive nodes	Mean (SD)	2.9 (3.6)	2.7 (4.0)	3.1 (3.1)	0.8
Duration of drains (days)	Mean (SD)	29.4 (17.8)	29.7 (16.6)	29.1 (19.6)	> 0.9
Adjuvant groin radiation	*N* (%)	6 (16%)	1 (5.0%)	5 (28%)	0.083
Seroma	*N* (%)	25 (66%)	15 (75%)	10 (56%)	0.3
Wound infection	*N* (%)	18 (47%)	9 (45%)	9 (50%)	> 0.9
Wound dehiscence	*N* (%)	7 (18%)	4 (20%)	3 (17%)	> 0.9

All but one patient underwent ilio-inguinal dissection, with no significant difference in the number of lymph nodes removed (mean 20.3, SD 7.0) or positive nodes (mean 2.9, SD 3.6) between groups. Drain duration was comparable (mean 29.4 days, SD 17.8). Adjuvant radiotherapy was more frequent in the CG-SLD group (28% versus 5%, *p* = 0.083). Postoperative complications were similar between groups. Seroma, following drain removal, occurred in 66% of patients, wound infection in 47%, and wound dehiscence in 18% (Table 2).

**Table 2:** Lymphedema incidence by time point

Cumulative rate of first lymphedema		Overall *N* = 38 [95% CI]	SC *N* = 20 [95% CI]	CG-SLD *N* = 18 [95% CI]	*p*
3 months	No	27 (71.1%) [54.1%, 84.6%]	11 (55.0%) [31.5%, 76.9%]	16 (88.9%) [65.3%, 98.6%]	0.033
	Yes	11 (28.9%) [15.4%, 45.9%]	9 (45.0%) [23.1%, 68.5%]	2 (11.1%) [1.4%, 34.7%]	
6 months	No	25 (65.8%) [48.6%, 80.4%]	10 (50.0%) [27.2%, 72.8%]	15 (83.3%) [58.6%, 96.4%]	0.043
	Yes	13 (34.2%) [19.6%, 51.4%]	10 (50.0%) [27.2%, 72.8%]	3 (16.7%) [3.6%, 41.4%]	
9 months	No	22 (57.9%) [40.8%, 73.7%]	9 (45.0%) [23.1%, 68.5%]	13 (72.2%) [46.5%, 90.3%]	0.11
	Yes	16 (42.1%) [26.3%, 59.2%]	11 (55.0%) [31.5%, 76.9%]	5 (27.8%) [9.7%, 53.5%]	
12 months	No	20 (52.6%) [35.8%, 69.0%]	9 (45.0%) [23.1%, 68.5%]	11 (61.1%) [35.7%, 82.7%]	0.4
	Yes	18 (47.4%) [31.0%, 64.2%]	11 (55.0%) [31.5%, 76.9%]	7 (38.9%) [17.3%, 64.3%]	
15 months	No	20 (52.6%) [35.8%, 69.0%]	9 (45.0%) [23.1%, 68.5%]	11 (61.1%) [35.7%, 82.7%]	0.4
	Yes	18 (47.4%) [31.0%, 64.2%]	11 (55.0%) [31.5%, 76.9%]	7 (38.9%) [17.3%, 64.3%]	
18 months	No	20 (52.6%) [35.8%, 69.0%]	9 (45.0%) [23.1%, 68.5%]	11 (61.1%) [35.7%, 82.7%]	0.4
	Yes	18 (47.4%) [31.0%, 64.2%]	11 (55.0%) [31.5%, 76.9%]	7 (38.9%) [17.3%, 64.3%]	
21 months	No	20 (52.6%) [35.8%, 69.0%]	9 (45.0%) [23.1%, 68.5%]	11 (61.1%) [35.7%, 82.7%]	0.4
	Yes	18 (47.4%) [31.0%, 64.2%]	11 (55.0%) [31.5%, 76.9%]	7 (38.9%) [17.3%, 64.3%]	
24 months	No	20 (52.6%) [35.8%, 69.0%]	9 (45.0%) [23.1%, 68.5%]	11 (61.1%) [35.7%, 82.7%]	0.4
	Yes	18 (47.4%) [31.0%, 64.2%]	11 (55.0%) [31.5%, 76.9%]	7 (38.9%) [17.3%, 64.3%]	

Within 24 months, 53% of patients experienced recurrence or death. Recurrence occurred in 40% of patients undergoing SC and 61% of patients undergoing CG-SLD. Death occurred in 35% and 33%, respectively. Distant recurrence was the most common pattern (71% overall).

### Primary End Point

Lymphedema Incidence

**Fig. 2:**
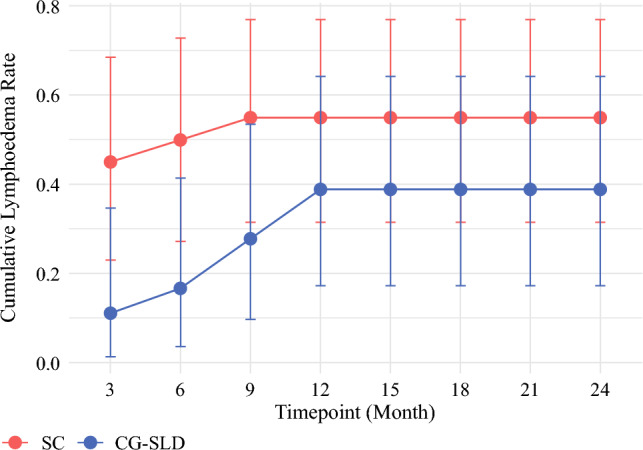
Lymphedema incidence by time point

At 24 months, the proportion of participants who presented with lymphedema (at least once) during follow-up was 47.4% (31.0–64.2), affecting a higher number of SC than CG-SLD participants: 55% (31.5–76.9) versus 38.9% (17.3–64.3) (*p* = 0.4) (Fig. 2). At 3 and 6 months, lymphedema incidence was significantly lower in the CG-SLD group; SC 45% (23.1–68.5) versus CG-SLD 11.1% (1.4–34.7) (*p* = 0.033) and SC 50% (27.2–72.8) versus CG-SLD 16.7% (3.6–41.4) (*p* = 0.043) respectively. By 9 and 12 months, between-group differences had narrowed and no new cases were recorded after 12 months

### Secondary End Points

#### Time to Lymphedema Development

**Fig. 3:**
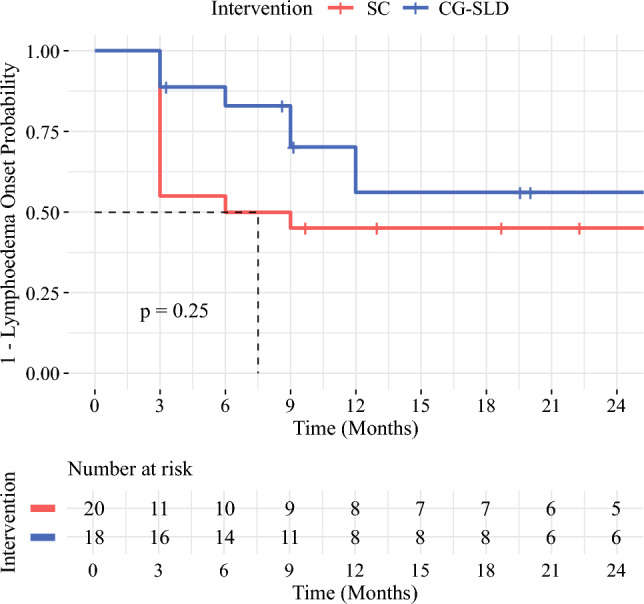
Kaplan–Meier estimates of lymphedema-free probability

Within the first year, Kaplan–Meier analysis demonstrated a higher probability of remaining lymphedema-free in the CG-SLD group compared with the SC group (56.2% versus 45.0% at 12 months) (Fig. 3). When accounting for death as a competing risk, the 12-month cumulative incidence of lymphedema was 55% in the SC group and 39% in the CG-SLD group, although this difference was not statistically significant (Gray’s test *p* = 0.20).

Lymphedema Severity

**Fig. 4:**
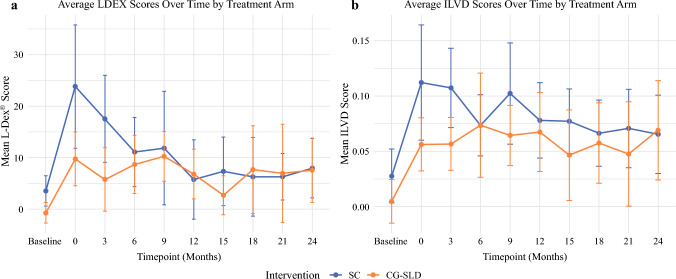
(**a** and **b**) Lymphedema severity

Lymphedema severity was numerically lower in the CG-SLD group within the first 6 months of follow-up. At 3 months, there was statistical evidence of a between-group difference. Mean (SD) L-Dex® was 5.8 (11.6) in the CG-SLD group compared with 17.5 (15.2) for the SC group, *p* = 0.024. Similarly, mean (SD) ILVD was 0.06 (0.10) in CG-SLD, which was lower than 0.11 (0.10) for the SC group (*p* = 0.019). These differences were not sustained at later time points (Fig. 4a and b).

#### Quality of Life

There were no between-group differences in EORTC or LYMQOL domains at any time point (Supplementary file). Although confidence intervals overlapped, between-group differences in EORTC global QoL met MID at several time points. Notably, the SC group had lower global QoL preoperatively (SC 65.0, CG-SLD 80.0) but higher scores at 15 (SC 75.0, CG-SLD 63.3) and 24 months (SC 76.7, CG-SLD 66.7).

At baseline, the CG-SLD group demonstrated higher mean physical functioning relative to SC meeting MID (SC 87.7, CG-SLD 97). Only 6% of CG-SLD and 16% of SC participants exceeded the TCI threshold at this time (Table 3). By 24 months, between-group MID had resolved (SC 86.7, CG-SLD 84.0), while the proportion exceeding TCI increased to 40% in both groups. Role functioning showed a similar pattern to global QoL: SC participants had lower preoperative mean scores exceeding MID (SC 84.2, CG-SLD 92.6), with three SC and zero CG-SLD participants exceeding TCI, but this trend reversed at 15 (SC 87.9, CG-SLD 75.9) and 24 months (SC 86.7, CG-SLD 75.0). At 24 months, no SC and two CG-SLD participants exceeded TCI for role functioning.

**Table 3:** Participants exceeding TCI in EORTC domains

EORTC domain (TCI^21^)		Time point *n*/*N* (%)								
		Pre-op	3 months	6 months	9 months	12 months	15 months	18 months	21 months	24 months
Physical function (< 83)	SC	3/19 (16%)	5/16 (31%)	6/14 (43%)	4/13 (31%)	1/11 (9.1%)	2/11 (18%)	4/11 (36%)	4/10 (40%)	4/10 (40%)
	CG-SLD	1/18 (5.6%)	2/16 (13%)	1/14 (7.1%)	2/12 (17%)	2/12 (17%)	2/9 (22%)	2/8 (25%)	1/7 (14%)	4/10 (40%)
Role function (< 58)	SC	3/19 (16%)	1/16 (6.3%)	0/14 (0%)	2/13 (15%)	1/11 (9.1%)	0/11 (0%)	1/11 (9.1%)	1/10 (10%)	0/10 (0%)
	CG-SLD	0/18 (0%)	2/16 (13%)	1/14 (7.1%)	1/12 (8.3%)	2/12 (17%)	2/9 (22%)	2/8 (25%)	0/7 (0%)	2/10 (20%)
Fatigue (> 39)	SC	3/19 (16%)	1/16 (6.3%)	4/14 (29%)	3/13 (23%)	1/11 (9.1%)	0/11 (0%)	0/11 (0%)	0/10 (0%)	0/10 (0%)
	CG-SLD	1/18 (5.6%)	1/16 (6.3%)	4/14 (29%)	1/12 (8.3%)	1/12 (8.3%)	3/9 (33%)	3/8 (38%)	2/7 (29%)	2/10 (20%)
Pain (> 25)	SC	4/19 (21%)	3/16 (19%)	4/14 (29%)	3/13 (23%)	2/11 (18%)	3/11 (27%)	3/11 (27%)	2/10 (20%)	2/10 (20%)
	CG-SLD	2/18 (11%)	5/16 (31%)	2/14 (14%)	1/12 (8.3%)	2/12 (17%)	3/9 (33%)	1/8 (13%)	3/7 (43%)	4/10 (40%)

Both groups started the study with a mean fatigue score of 16.7 but from 9 months onward, there was a trend toward greater fatigue in the CG-SLD group, such that by 24 months, mean fatigue was 13.3 in SC and 26.7 in CG-SLD groups, meeting MID. Elevated mean fatigue scores appeared to be driven by a small number of participants exceeding TCI during this period; beyond 12 months, no SC participants exceeded the fatigue TCI. Pain was numerically higher in the SC group preoperatively (SC 16.7, CG-SLD 6.7) but this was reversed and was higher in the CG-SLD group at 15 (SC 13.3, CG-SLD 23,3), 21 (SC 10.0, CG-SLD20.0), and 24 months (SC 6.7, CG-SLD 26.7). Both groups had three participants who exceeded the pain TCI at 15 months. At 24 months, more participants in the CG-SLD group reached pain TCI (*n* = 4, compared with SC *n* = 2).

LYMQOL domain scores (appearance, function, mood, and symptoms) remained low and stable in both groups over two years. Overall LYMQOL QoL scores followed a trajectory similar to EORTC global QoL.

## Discussion

This study demonstrates that a structured 6 month program of CG-SLD following groin dissection for metastatic melanoma may reduce the early incidence and severity of lower limb lymphedema. The benefit was evident at time points up to 6 months but was not sustained after cessation of the intervention. This time-dependent pattern suggests that the observed effect may be contingent on continued use of CG-SLD. However, the reduced sample size at later follow-up points likely limited statistical power to detect persistent differences.

These findings are consistent with evidence from breast cancer-related lymphedema prevention studies, where early compression therapy and proactive surveillance was found to be beneficial in reducing lymphedema incidence.^10–12^ Those studies provided some evidence that prophylactic compression was able to reduce progression to chronic lymphedema. Early intervention is thought to promote lymphatic adaptation and limit extracellular fluid accumulation. Johansson et al.^12^, in a review of compression garment studies, supported the role of prophylactic compression for at-risk patients with breast cancer but highlighted the lack of high-quality randomized controlled trials.

Our findings suggest that CG-SLD can delay the onset of leg lymphedema in melanoma patients undergoing groin dissection. This is clinically meaningful, as early lymphedema is associated with greater symptom burden and increased risk of chronic progression.^23^ Limiting early fluid accumulation may reduce tissue distension and inflammatory changes that contribute to long-term lymphatic dysfunction. Longer duration of compression beyond 6 months, or targeted prophylaxis in high-risk patients (e.g., those with elevated BMI or receiving adjuvant radiotherapy)^24^, may be required to achieve sustained benefit.

We acknowledge that the higher proportion of patients had adjuvant radiotherapy in the CG-SLD group and that this may have biased results toward increased lymphedema risk in that group. The early reduction observed despite this imbalance may support the possibility of a treatment effect; however, small sample size precluded robust multivariable adjustment.

Our overall lymphedema incidence of 47.4% (31.0–64.2) at 24 months is slightly higher compared with previously reported rates of 25–42% following groin dissection.^5–7^ The persistence of moderate rates in both groups likely reflects the extensive nature of groin dissections and the high baseline risk in this population. As reported in prior meta-analyses and prospective studies, variability in incidence across studies is largely attributable to differences in definitions, measurement tools, and follow-up duration^5^. The use of both bioimpedance and volumetric criteria in our study provided a sensitive and objective assessment of lymphedema progression, consistent with International Society of Lymphology recommendations.^4^

CG-SLD was formally delivered and monitored during the 6 months after surgery only, although some participants continued the interventions thereafter. The trend towards higher fatigue, pain, and lower QoL scores in the CG-SLD group beyond 9 months raises the possibility that ongoing self-management strategies may have imposed a degree of burden for some patients. Compression garments can affect comfort, body image, and daily functioning, and the requirement for regular application and maintenance can be intrusive.^25^ In cancer survivors already coping with the physical and psychological sequelae of melanoma treatment, the addition of sustained self-directed lymphatic care may contribute to fatigue. However, these findings must be interpreted cautiously. Mean differences were frequently influenced by small numbers of participants exceeding the TCI threshold, and confidence intervals overlapped between groups. It is equally plausible that those with emerging symptoms were more likely to persist with CG-SLD.

## Limitations

This study enrolled a particularly high-risk melanoma population, during an era prior to the widespread adoption of effective modern systemic melanoma therapies. As a result, mortality and disease recurrence were common and substantially reduced the number of patients available for long-term follow-up, particularly beyond 9 months. Consequently, the study was underpowered relative to the original sample size calculation, and the findings should be interpreted as exploratory and hypothesis-generating. Future adequately powered studies should incorporate the optimal duration and intensity of preventive compression strategies following groin dissection, as well as measures of treatment burden and patient experience to better understand the balance between potential lymphedema prevention and the psychosocial impact of prolonged self-management interventions.

We believe our results remain clinically relevant for patients who continue to require groin dissection, particularly in settings where contemporary systemic therapies are not readily accessible, or among patients who do not respond to systemic treatment and still require surgical management. In this context and given the decreasing number of patients now undergoing groin dissection worldwide, our data represent a valuable and potentially unique contribution to the evidence base in this area.

## Conclusions

CG-SLD was associated with a reduction in lower limb lymphedema during the first 6 months following groin dissection, corresponding to the period of active intervention. This effect was not sustained after cessation of compression, suggesting that early intervention may only delay lymphoedema. Larger, adequately powered studies are needed to determine whether longer duration prophylactic strategies can provide lasting benefit.

## Supplementary Information

Below is the link to the electronic supplementary material.

Supplementary file1 (DOCX 24 KB)
