# Supplementary material for: Minimizing Early-Onset Lymphedema Following Groin Dissection in Metastatic Melanoma
Source: Ann Surg Oncol. 2026 Apr 13;33(7):6737–45. doi: 10.1245/s10434-026-19559-4 (PMC13242378; doi:10.1245/s10434-026-19559-4)
Supplement: Supplementary file 1 — Supplementary file1 (DOCX 24 KB) [file 10434_2026_19559_MOESM1_ESM.docx]

**EORTC**

**Mean (CI*) scores by domain and timepoint**

| **Domain** | **Intervention** | **Pre-op** | **3 months** | **6 months** | **9 months** | **12 months** | **15 months** | **18 months** | **21 months** | **24 months** |
| --- | --- | --- | --- | --- | --- | --- | --- | --- | --- | --- |
| **Physical Function** | SC | 87.7 (76.0, 99.5) | 88.3 (82.8, 93.9) | 83.8 (73.3, 94.3) | 82.8 (68.5, 97.1) | 92.1 (85.2, 99.0) | 87.0 (75.6, 98.3) | 84.4 (72.6, 96.1) | 86.7 (76.6, 96.9) | 86.7 (77.7, 95.7) |
|  | CG-SLD | 97 (93.6, 100.0) | 91.7 (85, 98.4) | 91.8 (83.6, 99.9) | 93.9 (88.1, 99.7) | 90.6 (82.2, 99.1) | 89.6 (77.6, 100.0) | 90.8 (81, 100.0) | 90.5 (79.3, 100.0) | 84.0 (72.3, 95.7) |
| **Role Function** | SC | 84.2 (68.7, 99.7) | 85.4 (75.8, 95.1) | 85.7 (76.6, 94.8) | 82.0 (63.0, 100.0) | 86.4 (74.3, 98.4) | 87.9 (77.8, 98.0) | 86.4 (73.3, 99.4) | 88.3 (75.7, 100.0) | 86.7 (74.4, 99.0) |
|  | CG-SLD | 92.6 (86.7, 98.4) | 83.3 (72.6, 94.1) | 86.9 (75.5, 98.3) | 87.5 (76.3, 98.7) | 80.6 (64.4, 96.7) | 75.9 (54.6, 97.3) | 83.3 (61, 100.0) | 88.1 (73.5, 100.0) | 75.0 (55.3, 94.7) |
| **Fatigue** | SC | 16.7 (3.3, 30.0) | 16.7 (10.0, 26.7) | 20.0 (10.0, 33.3) | 23.3 (6.7, 40.0) | 13.3 (3.3, 23.3) | 13.3 (3.3, 20.0) | 16.7 (6.7, 23.3) | 10.0 (0, 16.7) | 13.3 (3.3, 23.3) |
|  | CG-SLD | 16.7 (10.0, 23.3) | 20.0 (13.3, 26.7) | 26.7 (10.0, 40.0) | 20.0 (13.3, 30.0) | 23.3 (16.7, 33.3) | 30.0 (16.7, 46.7) | 30.0 (6.7, 56.7) | 30.0 (6.7, 50.0) | 26.7 (10.0, 40.0) |
| **Pain** | SC | 16.7 (3.3, 30.0) | 10.0 (3.3, 20.0) | 13.3 (3.3, 23.3) | 13.3 (0.0, 30.0) | 13.3 (3.3, 20.0) | 13.3 (3.3, 23.3) | 13.3 (0.0, 26.7) | 10.0 (0.0, 26.7) | 6.7 (0.0, 20.0) |
|  | CG-SLD | 6.7 (0.0, 13.3) | 16.7 (3.3, 30.0) | 10.0 (0.0, 20.0) | 6.7 (0.0, 13.3) | 10.0 (0.0, 16.7) | 23.3 (0.0, 46.7) | 6.7 (0.0, 16.7) | 20.0 (10.0, 33.3) | 26.7 (3.3, 46.7) |
| **Global QoL** | SC | 65.0 (51.7, 78.3) | 68.3 (56.7, 78.3) | 76.7 (68.3, 85.0) | 76.7 (66.7, 86.7) | 83.3 (75.0, 93.3) | 75.0 (66.7, 85.0) | 76.7 (70.0, 83.3) | 75.0 (66.7, 83.3) | 76.7 (70.0, 83.3) |
|  | CG-SLD | 80.0 (73.3, 88.3) | 75.0 (65.0, 83.3) | 70.0 (58.3, 83.3) | 81.7 (75.0, 90.0) | 76.7 (63.3, 90.0) | 63.3 (45.0, 80.0) | 70.0 (55.0, 86.7) | 68.3 (56.7, 81.7) | 66.7 (51.7, 80.0) |

*Confidence intervals were truncated to the possible score range (0, 100). Higher scores indicate better QoL or function, whereas for symptom domains, lower scores indicate fewer symptoms.

**LYMQOL**

**Mean (CI) by domain and timepoint**

| **Domain** | **Intervention** | **Pre-op** | **3 months** | **6 months** | **9 months** | **12 months** | **15 months** | **18 months** | **21 months** | **24 months** |
| --- | --- | --- | --- | --- | --- | --- | --- | --- | --- | --- |
| **Appearance** | SC | 1.2 (0.9, 1.5) | 1.4 (1.2, 1.7) | 1.5 (1.2, 1.9) | 1.5 (1.1, 1.9) | 1.5 (1.1, 1.9) | 1.6 (1.1, 2.1) | 1.5 (1.1, 2) | 1.5 (1, 2) | 1.4 (1, 1.8) |
|  | CG-SLD | 1 (1, 1) | 1.1 (1, 1.2) | 1.1 (1, 1.3) | 1.1 (1, 1.2) | 1.1 (1, 1.3) | 1.1 (0.9, 1.4) | 1.1 (1, 1.2) | 1.1 (0.9, 1.3) | 1.1 (1, 1.2) |
| **Function** | SC | 1.2 (0.9, 1.4) | 1.6 (1.3, 1.9) | 1.5 (1.2, 1.9) | 1.6 (1.2, 2.1) | 1.3 (1, 1.6) | 1.4 (1.1, 1.8) | 1.4 (1, 1.9) | 1.3 (0.9, 1.7) | 1.3 (1, 1.5) |
|  | CG-SLD | 1.1 (0.9, 1.2) | 1.3 (1.1, 1.5) | 1.3 (1, 1.5) | 1.3 (1.1, 1.5) | 1.5 (1, 2) | 1.3 (0.8, 1.7) | 1.3 (1.1, 1.6) | 1.3 (1, 1.7) | 1.3 (0.9, 1.7) |
| **Mood** | SC | 1.7 (1.3, 2.2) | 1.4 (1.2, 1.7) | 1.4 (1.1, 1.7) | 1.6 (1.2, 2) | 1.4 (1, 1.7) | 1.4 (1.1, 1.8) | 1.4 (1.1, 1.8) | 1.2 (1, 1.4) | 1.4 (1, 1.7) |
|  | CG-SLD | 1.4 (1.1, 1.7) | 1.6 (1.2, 2) | 1.9 (1.4, 2.4) | 1.3 (1.1, 1.5) | 1.5 (1.1, 1.9) | 1.6 (1.1, 2.1) | 1.7 (1, 2.3) | 1.5 (1, 2) | 1.7 (1.2, 2.3) |
| **Symptoms** | SC | 1.2 (0.9, 1.4) | 1.5 (1.2, 1.8) | 1.5 (1.2, 1.8) | 1.7 (1.2, 2.2) | 1.5 (1.2, 1.8) | 1.5 (1.2, 1.9) | 1.6 (1.2, 1.9) | 1.4 (1.1, 1.8) | 1.3 (1.1, 1.5) |
|  | CG-SLD | 1 (1, 1.1) | 1.4 (1.3, 1.6) | 1.5 (1.3, 1.8) | 1.4 (1.1, 1.6) | 1.6 (1.2, 2) | 1.6 (1, 2.2) | 1.5 (1, 2) | 1.4 (1, 1.8) | 1.4 (0.9, 2) |
| **Overall QoL** | SC | 6.7 (5.5, 7.9) | 7.1 (6, 8.3) | 7.8 (6.9, 8.7) | 6.9 (5.9, 8) | 8.3 (7.2, 9.4) | 7.6 (6.4, 8.8) | 7.6 (6.6, 8.6) | 7.6 (6.5, 8.6) | 7.1 (5.4, 8.9) |
|  | CG-SLD | 8.1 (7.3, 8.9) | 7.3 (6.4, 8.2) | 6.8 (5.8, 7.8) | 7.4 (6.8, 8.1) | 7.2 (5.9, 8.6) | 7 (5.4, 8.6) | 6.9 (5.1, 8.6) | 7.3 (5.5, 9.2) | 7.2 (5.9, 8.5) |

Function, appearance, symptoms and mood ranged from 1 to 4, where lower scores indicate fewer problems. Global QoL score ranged from 0 to 10, with higher scores indicating better QoL.
